# Middlesex Hospital

**Published:** 1830-08

**Authors:** 


### HOSPITAL REPORTS.

### MIDDLESEX HOSPITAL.

### *Removal of a large fatty Tumor.*

Henry Hoare, æt. sixty, was admitted into the hospital on the 19th of June, under the care of Mr. Mayo. His complaint was a large tumor, attached to and covering the right hip: it had been growing thirty years, and, except through its weight, had given no inconvenience till recently, when part of the surface had inflamed and ulcerated. The tumor was easily moveable upon the crest of the ilium and the glutei muscles, on which it rested. It presented the soft elastic lobulated character of a fatty tumor, which, upon examination, it proved to be.

The tumor was removed by Mr. Mayo on the 14th of June: it weighed thirteen pounds two ounces. Enough of the integument had been dissected from the tumor to allow the edges of the skin to be brought together and held in contact with sutures, and strips of adhesive plaster. The wound went on favorably: the greater part of the skin united to the muscle below by adhesion; the patient had entirely recovered in five weeks, a long and narrow cicatrix upon the hip alone remaining. About sixteen ounces of blood were lost in the operation, which occupied scarcely more than half a minute, and about a dozen small arteries required to be tied. Two or three remarkably large venous trunks round the base of the tumor were divided in the incision through the integuments, but, after the first gush of blood, no more flowed from them.

---

### *Operation of Lithotomy, to remove part of a Catheter from the Bladder.*

John Mitchell, ætat. thirty, was admitted into this hospital, under the care of Mr. Mayo, on the 20th of April. Three weeks before this, being under treatment for stricture, a surgeon, who attended him, passed a small elastic catheter into the bladder, which broke, leaving five inches behind. Between the period of this accident and his admission, the urethra had been dilated by the passage of instruments, and various attempts had been made, by means of bladder forceps, to extract the broken catheter.

At the period of his admission, this patient suffered only, when in voiding his urine, the bladder became nearly empty: the expulsion of the last drops of urine was attended with pain in the bladder, which lasted several minutes. The urine, however, was perfectly clear, and there was no call to pass it oftener than usual.

Upon examining the bladder with a sound, neither Mr. Mayo nor his colleagues could detect the presence of the broken catheter. Mr. Mayo, however, believing, from the history and symptoms of the case, that the foreign body must still be there, made several attempts with various instruments to seize it, and draw it through the urethra: these attempts were entirely fruitless, and brought on a slight degree of fever; they were therefore discontinued. With the use of opiates, the warm bath, and alkaline drinks, the patient's condition speedily improved. The feverish symptoms left him, the pain on voiding the urine lessened, and sometimes for a day he was wholly free from it.

After a time, however, the symptoms became aggravated; the pain in the bladder on passing the urine was increased; pain of the same description was felt upon evacuating the bowels; the urine became turbid, and there adhered to the bottom of the vessel a viscid puriform mucus. The patient now passed fragments of calcareous matter, to which small portions of the crust of the catheter adhered. Under these circumstances, Mr. Mayo examined the bladder anew with the sound, determining to make fresh attempts, if any thing should be felt in the bladder, to extract it through the urethra. But the lining membrane of the bladder was now acutely sensible, and the presence of the sound could scarcely be borne by the patient. Portions of calcareous matter were, however, distinctly felt in the bladder; and the patient expressed a strong wish to be cut, rather than to suffer renewed attempts at extraction by the urethra.

Accordingly, on the 12th of July, the lateral operation was performed by Mr. Mayo. The bladder being cut into, the broken catheter was taken out in two portions, each having a thick calcareous concretion upon it. This concretion was of a brittle nature and some difficulty was experienced in clearing the bladder of the fragments that were detached; the bladder was washed out with warm water, but still some fragments remained. The patient, who appeared to suffer considerable pain from the process, was then returned to bed, and forty drops of laudanum were administered to him. Three hours afterwards, some urine having flowed away, not containing calcareous matter, Mr. Mayo again introduced the tube of a syringe into the bladder, and injected warm water. A slight attack of shivering followed, which recurred early the following morning; but the bowels being freely moved in the course of the day, the patient felt relieved. His subsequent progress has been perfectly favorable, and several fragments of calcareous matter, probably all that remained, have flowed away in the urine.

Mr. Mayo employed a double-edged scalpel and Blizard's knife, and remarked, in reference to the operation of lithotomy, and to the instruments used in it: 1st, that the outward wound should be very oblique, by which means its direction is brought to the nearest correspondence with that of the incision in the bladder, and the rectum is placed in the least danger of being wounded; 2d, that the groove in the staff should be opened towards the left side, by making the left edge of the groove considerably shallower than the right; 3d, that the curve of the staff should be sudden, and that the part beyond the curve should be of sufficient length, and nearly straight, not bent upwards at the point; 4, that the groove in the staff should terminate abruptly, and a full third of an inch from the end of the staff; 5, that the scalpel employed to cut into the urethra and bladder should have a double edge, in order that it may be the easier run into the groove of the staff; and that the upper surface of the scalpel itself is all the better for having an oblique, shallow groove, corresponding with the left edge of the groove of the staff; 6, that the Blizard's knife, which Mr. Mayo recommends, if the operator does not employ the scalpel to complete the incision, should have a short cutting edge, and should end in a large square beak, calculated to strike full against, and to be securely checked by the deep, square, and abrupt termination of the groove of the staff, the beak being set upon the blade just so obliquely as to give the edge the requisite direction downward and outwards; for moving in which direction, the Blizard's knife, it may be observed, is already disengaged from the staff, by the joint effect of the obliquity of the beak and of the shallowness of the left edge of the groove of the staff. A skilful hand will no doubt execute this operation well with any instrument, but it must be desirable to adapt instruments as exactly as possible to the objects upon which they are to be employed.

The subject of this case bore the operation with much less than ordinary fortitude; and it is but an act of justice to Mr. Mayo to express our admiration of his perfect self-possession and operative dexterity with so unruly a patient.

